# The Global State of the Genetic Counseling Profession

**DOI:** 10.1038/s41431-018-0252-x

**Published:** 2018-10-05

**Authors:** MaryAnn Abacan, Lamia Alsubaie, Kristine Barlow-Stewart, Beppy Caanen, Christophe Cordier, Eliza Courtney, Emeline Davoine, Janice Edwards, Niby J. Elackatt, Kate Gardiner, Yue Guan, Lian-Hua Huang, Charlotta Ingvoldstad Malmgren, Sahil Kejriwal, Hyon J. Kim, Deborah Lambert, Paulina Araceli Lantigua-Cruz, Juliana M. H. Lee, Marianne Lodahl, Åshild Lunde, Shelley Macaulay, Ivan Macciocca, Sonia Margarit, Anna Middleton, Ramona Moldovan, Joanne Ngeow, Alexandra J. Obregon-Tito, Kelly E. Ormond, Milena Paneque, Karen Powell, Kunal Sanghavi, Diana Scotcher, Jenna Scott, Clara Serra Juhé, Shiri Shkedi-Rafid, Tina-Marié Wessels, Sook-Yee Yoon, Catherine Wicklund

**Affiliations:** 1 Institute of Human Genetics, National Institutes of Health, University of the Philippines Manila, Manila, Philippines; 2 King Abdullah Specialized Children’s Hospital (KASCH), King Abdulaziz Medical City, Riyadh, Saudi Arabia; 3 Northern Clinical School, Faculty of Medicine and Health, University of Sydney, Sydney, NSW Australia; 4 Department of Clinical Genetics, Maastricht University Medical Centre, Maastricht, The Netherlands; 5 Department of Genetics, SYNLAB Genetics, Lausanne, Switzerland; 6 Cancer Genetics Service, Division of Medical Oncology, National Cancer Centre, Singapore, Singapore; 7 Lausanne University Hospital (CHUV), Lausanne, Switzerland; 8 Transnational Alliance for Genetic Counseling, University of South Carolina Genetic Counseling Program, University of South Carolina, Columbia, SC USA; 9 Organization for Rare Diseases India, Cloudnine Hospitals, Bangalore, India; 10 LifeLabs Genetics, Toronto, ON Canada; 11 Rollins School of Public Health, Emory University, Atlanta, GA USA; 12 School of Nursing, China Medical University, Taichung, Taiwan; 13 School of Nursing, College of Medicine, National Taiwan University, Taipei, Taiwan; 14 Center for Fetal Medicine and Clinical Genetics, Karolinska University Hospital, Stockholm, Sweden; 15 Department of Clinical Science, Intervention and Technology, Karolinska Institutet, Stockholm, Sweden; 16 Department of Public Health and Caring Science, Uppsala University, Uppsala, Sweden; 17 Department of Womenʼs and Childrenʼs Health, Uppsala University, Uppsala, Sweden; 18 Institute for Public Health Genetics, University of Washington, Seattle, USA; 19 Ajou Univ. Medical School and Konyang Univ. Graduate school, Yeongtong-gu, Suwon, South Korea; 20 National Rare Diseases Office, Dublin, Ireland; 21 University of Medical Sciences of Havana, Havana, Cuba; 22 National University of Malaysia, Kuala Lumpur, Malaysia; 23 Department of Clinical Genetics Rigshospitalet, Copenhagen University Hospital, Copenhagen, Denmark; 24 Department of Global Public Health and Primary Care, University of Bergen, Bergen, Norway; 25 Division of Human Genetics, Faculty of Health Sciences, University of the Witwatersrand & The National Health Laboratory Service, Johannesburg, South Africa; 26 Victorian Clinical Genetics Services, Melbourne, Australia; 27 Clínica Alemana Universidad del Desarrollo, Facultad de Medicina, Centro de Genética y Genómica, Santiago, Chile; 28 Society and Ethics Research, Connecting Science, Wellcome Genome Campus, Cambridge, UK; 29 Faculty of Education, University of Cambridge, Cambridge, UK; 30 Department of Psychology, Babeș-Bolyai University, Cluj-Napoca, Romania; 31 University of Arkansas for Medical Sciences, Little Rock, AR USA; 32 Department of Genetics and Stanford Center for Biomedical Ethics, Stanford University School of Medicine, Stanford, CA USA; 33 Stanford University School of Medicine, 300 Pasteur Drive, MC 5208 Stanford, CA USA; 34 i3S – Instituto de Investigação e Inovação em Saúde, CGPP - Centre for Predictive and Preventive Genetics and IBMC – Institute for Molecular and Cell Biology, Universidade do Porto, Porto, Portugal; 35 Cone Health Cancer Center, Greensboro, NC USA; 36 The Jackson Laboratory for Genomic Medicine, Farmington, CT USA; 37 Manchester Centre for Genomic Medicine, Manchester University Hospitals NHS Foundation Trust, Saint Mary’s Hospital, Manchester, UK; 38 University of British Columbia, Vancouver, BC Canada; 39 Departament de Ciències Experimentals i de la Salut, Universitat Pompeu Fabra, Institut Hospital del Mar d’Investigacions Mèdiques, Centro de Investigación Biomédica en Red de Enfermedades Raras, Barcelona, Spain; 40 Hadassah Hebrew University Medical Center, Jerusalem, Israel; 41 Division Human Genetics, University of Cape Town, Cape Town, South Africa; 42 Cancer Research, Subang Jaya, Malaysia; 43 University Malaya Medical Centre, Kuala Lumpur, Malaysia; 44 National University of Malaysia, Kuala Lumpur, Malaysia; 45 Feinberg School of Medicine, Northwestern University, Chicago, IL USA

**Keywords:** Genetic counselling, Genetic services

## Abstract

The profession of genetic counseling (also called genetic counselling in many countries) began nearly 50 years ago in the United States, and has grown internationally in the past 30 years. While there have been many papers describing the profession of genetic counseling in individual countries or regions, data remains incomplete and has been published in diverse journals with limited access. As a result of the 2016 Transnational Alliance of Genetic Counseling (TAGC) conference in Barcelona, Spain, and the 2017 World Congress of Genetic Counselling in the UK, we endeavor to describe as fully as possible the global state of genetic counseling as a profession. We estimate that in 2018 there are nearly 7000 genetic counselors with the profession established or developing in no less than 28 countries.

## Introduction

“A genetic counselor is like air conditioning. When you do not have it, you do not realize you are missing it, but when you have it, you cannot live without it!” (paraphrased from a French Geneticist, as presented at TAGC 2016 by E. Davoine)

While the service of genetic counseling was in existence much earlier, the profession of genetic counseling (called genetic counselling in many countries, but shortened in this paper for consistency) began in the United States (US) in 1969 [1], approaching 50 years ago. Global expansion of genetic counseling in the early 1990’s [2, 3] led to the transition from genetic counseling being solely provided as part of the role of medical physicians (often, but not always medical geneticists) towards an international allied health profession dealing with inherited conditions. As defined by the National Society of Genetic Counselors in the US (NSGC, 2006), “Genetic counseling is the process of helping people understand and adapt to the medical, psychological and familial implications of genetic contributions to disease. This process integrates the following: (1) Interpretation of family and medical histories to assess the chance of disease occurrence or recurrence. (2) Education about inheritance, testing, management, prevention, resources and research. (3) Counseling to promote informed choices and adaptation to the risk or condition.” [4] While the role of genetic counselors (GCs) most consistently includes the clinical roles as described in the NSGC definition, many GCs now also work in research, laboratory, industry, educational, policy, and advocacy positions in many locations, and the scope of clinical practice varies both within and between countries. The commonality of genetic counseling as a profession is that individuals work primarily in the area of heritable disorders and related genetic testing, providing education and emotional support to patients and families who are impacted by them. As of 2018, genetic counseling is a healthcare profession with a presence in nearly all medical specialties, most frequently obstetrics, pediatrics (dealing with rare diseases), oncology, cardiology, and neurology.

The international expansion of genetic counseling has been a topic of interest for the past decade. The Transnational Alliance of Genetic Counseling (TAGC) was established by Janice Edwards (USC) via a Jane Engleberg Memorial Fellowship through NSGC in 2006 [5]. TAGC fosters communication within the international genetic counseling community, specifically with a focus on genetic counseling education transnationally. TAGC has a website and listserv, and has hosted international meetings in 2006 (Manchester), 2008 (Barcelona), 2011 (Montreal), and 2016 (Barcelona). The year 2017 also saw the first World Congress of Genetic Counselling in Cambridge, UK, hosted by Connecting Science at the Wellcome Genome Campus. This World Congress had over 220 GC attendees from 24 different countries and focused on empirical research on the genetic counselling process.

Through these international meetings, it became clear that while there are conceptually many similarities in approach to practice, there are different training and practice models globally. Some differences are based on the variations in healthcare systems, regulatory systems (see, for example, http://www.eurogentest.org/index.php?id=679, accessed May 5, 2018), and university level training that is available in different countries. Examples of these differences include: the length of training, specific curriculum, and amount of clinical training; the background of individuals who are recruited into the profession (for example training nurses, midwives, or laboratory geneticists) (see, for example, a European review [6]) and the manner in which practicing providers are certified, registered, and/or licensed. For the purposes of this paper, since the genetic counseling profession was first developed in the US and many countries modeled the development of the profession on the US’s scope of practice and related training standards, these will be used as the basis of comparison when describing genetic counselor practice globally.

Several summaries exist detailing genetic counseling in specific countries or regions, including a special edition of the *Journal of Genetic Counseling* in December 2013 (volume 22:6), with many papers from that edition referenced for readers who desire more detailed information, and a commentary by some co-authors of this paper [7]. A comprehensive summary of the genetic counseling profession is presented below. It is primarily based on information presented at the 2016 TAGC meeting in Barcelona, with supplemental information added by those presenters and by others identified through TAGC, the NSGC International SIG and attendance at the 2017 World Congress of Genetic Counselling. We strive to be as internationally complete as possible and to cite publicly available references for the data presented in this paper, but in the event we have missed countries where genetic counseling is in development, we invite representatives from those countries to contact TAGC and become connected to the global community of GCs.

**Fig. 1:**
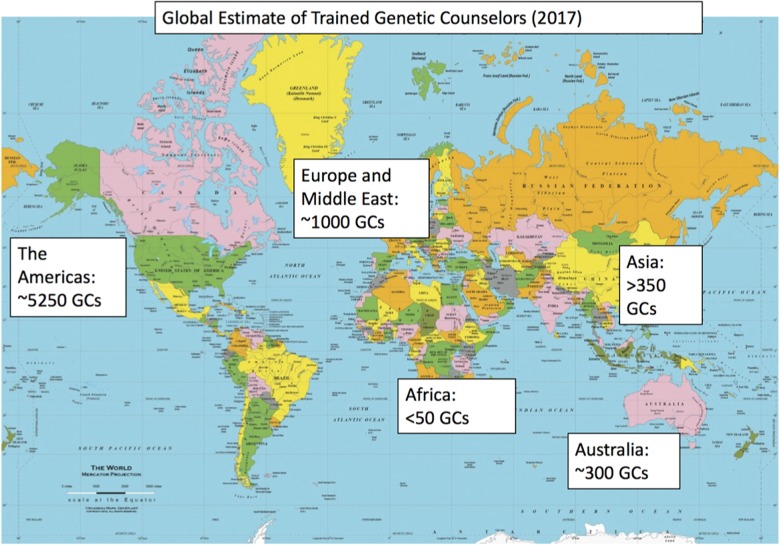
Map of genetic counselors internationally (2017)

**Fig. 2:**
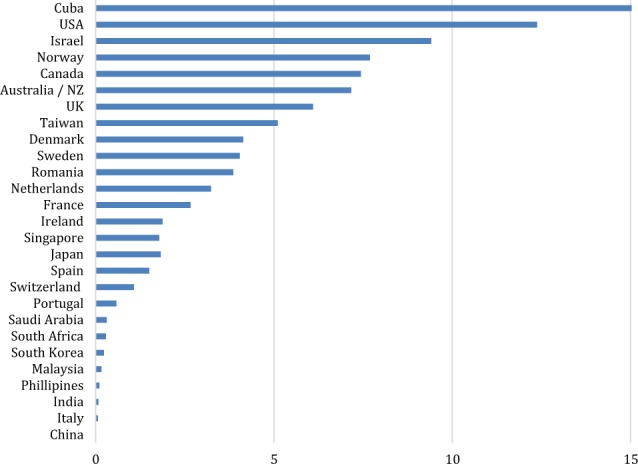
Trained genetic counselors per million population based on country population and estimated number of genetic counselors. (Assumes all trained genetic counselors are practicing clinically, which may significantly overestimate calculations in several countries, particularly the US and Canada where 1/3–1/4 do not regularly see patients. Cuba data is >75/million). Only a handful of countries come close to achieving the workforce recommendation, made by the Royal College of Physicians UK to have 6–12 genetic counselors/million population [48].

The majority of data for this paper is presented in tables, with the text elaborating on specifics that are unique to a particular country. Table 1 summarizes details of the numbers of GCs, training, and regulatory approaches across the globe; it is based on self-reported data from the paper authors, relevant publications, and publicly available websites including those of professional organizations. Table 2 outlines the requirements for credentialing (certification, registration) globally. Finally, Table 3 references the various professional organizations, defines their acronyms, and lists relevant websites for additional information and documentation of much of the data presented in the paper. We also provide images of the global distribution of genetic counseling (Fig. 1) and the ratio of GCs per million of population (Fig. 2) based on the data presented in Table 1.

**Table 1:** State of genetic counseling globally

Country	Total population estimate	Estimated number of GCs	Year first training program started	Training programs and degree	Number in training/year	Is there national regulation	Is there state/ province regulation	Is there professional body regulation (certification or registration body)
United States of America	~327 million	~4000	1969	39 (MS)	~400/yr	No	Yes (20+ states with licensure)	Yes (ABGC)
Canada	~37 million	~350	1985	5 (MS)	20–25/yr	No	No	Yes (CAGC and ABGC)
Cuba	~11 million	~900	1999	1 (MS)	50 every other year	No	No	No
UK	~60 million	~310	1992	3 (MS → STP)	~40/yr	As of 2018, (HCPC) to graduates of the 3-year STP. National voluntary regulation via GCRB for others	No	Yes (GCRB)
Denmark	~5.8 million	24	—	—	—	No	No	No
France	~66.9 million	>175	2004	1 (MS)	20/yr	No	No	Yes (EBMG)
Ireland	4.8 million	9	—	—	—	No	No	No
The Netherlands	17 million	55	1992; 2017 (MPA)	4 (MPA/GC)	8	No	No	No
Norway	5 million	40	2001	1 (MS)	10 every other yr	Yes (Biotechnology Act)	No	No
Portugal	10 million	6 trained (3 working as GCs)	2009	1 (not currently active)	6	No	No	No
Romania	20 million	76 trained	2009	1 (MS; not currently active)	13–17	No	No	Yes (EBMG)
Spain	46 million	70	2008	1 (MS)	10–12 every other yr	No	No	Yes (EBMG)
Sweden	10 million	30	—	—	—	No	No	Yes (SFMG)
Switzerland	2.1 million French cantons; 8.3 million total	~10 (primarily French cantons)	—	—	—	No	No	No
Israel	8.5 million	80	1997	3 (MS)	20/yr	Yes (Ministry of Health)	No	No
Saudi Arabia	32 million	20	2005 (GradDip); 2015 (MS)	2 (MS)	~10/yr	Yes (SCFHS)	No	No
Australia/ New Zealand	~30 million combined	220	1995 (GradDip); 2011 (MS)	2 (MS)	40/yr	In progress (self-regulation)	No	Yes (HGSA)
China	~1.34 billion	Unknown	—	—	—	No	No	Yes (CBGC)
India	~1.324 Billion	~76	2007 (Cert.) 2014 (MS)	1(MS) 1(GradDip) 2(Cert.)	unknown	No	No	Yes (IBGC)
Japan	126 million	~230	2000s	14 (MS)	Unknown	No	No	Yes (JGC)
Malaysia	32 million	5	2015	1	1–4/yr	No	No	No
Philippines	100 million	11	2010	1 (MS)	4–10/yr	No	No	No
Singapore	5.6 million	10	—	—	—	No	No	No
South Korea	~50 million	12	2006	Unknown	Unknown	No	No	No
Taiwan	~23 million	120	2003	1 (MS)	13/yr	No	No	Yes (TAGC)
South Africa	57 million	20	1989	2 (MS)	2–5/yr	Yes (HPCSA)	No	No (GC-SA, through SASHG)

**Table 2:** Requirements for credentialing

Country	Type of regulation (body)	Requirements	Written exam?	Oral exam?	Recertification
United States of America	Certification (ABGC)	Graduation from ABGC approved accredited program. 50 case logbook.	Yes, offered twice yearly. 3 attempts possible.	No	Q 5 years by continuing education or exam.
Canada	Certification (CAGC)	Graduation from approved MS program; 50 case logbook; letters of recommendation.	Yes, offered every 2 years. 2 attempts possible. English/French	No	Q 10 years by: professional work experience and continuing education; or exam.
UK	Registration (GCRB)	Approved 2-year MS + 2 years post-graduate work experience in RCGS. Portfolio with 50 cases, references, essay, 3 case studies (ethical, counseling, and scientific reflection), evidence of counseling supervision, recorded consultations and professional development reflection.	No	No	Q 5 years
Europe	Registration (EBMG)	Approved MS program + 2 years post-graduate work; reflective essays and case studies; case logbook; references.	Grandfather Clause C only	No	Q 5 years
Israel	Licensure (Israeli Ministry of Health)	2 years post-graduate work; 85 case logbook. Certification given 1 year after exam if working in genetics clinic and receive recommendation from clinic head.	Yes	No	Permanent after 1-year post-exam with rec.
Saudi Arabia	Licensure (SCFHS)	Panel interview, testing, or exam by a medical board	No	Yes	Under review
Australia (includes New Zealand)	Certification (HGSA)	Minimum 2 years of practice post-graduate in a service that can provide supervision; 50 case logbook; 5 long case studies; reflective practice essay; literature review or first author publication. Assessed simulated consultation and interview; training/supervisor reports.	No	No	Under review but currently voluntary Q 5 years; requires continuing professional development
India	Certification (BCG);	Level 1 – MS Life Sc. + 1-year course/experience in Genetic Counseling; observe at least 100 GC. Logbook (20 cases) + 2 case studies + reflective essays Level 2–5+ years and at least 500 cases Log cases (50) + Long case study (5) + reflective essays (2)	Yes (offered yearly)	Yes	Q 2–3 years; requires continuing education or professional activities
Japan	Certification (JBGC)	Graduation from approved program; active membership in JSHG or JSGC for 2 years	Yes	Yes	Q 5 years by continuing education
Taiwan	Certification (TAGC)	Based on a combination of (1). Educational background (20%), (2) School transcript or certification from work (10%) (3). Work experience (10%) (4). 50 genetic counseling case log record and (5) 10 paper records and 50 electronic records (60%)	Yes	Yes	Q6 years by continuing education
South Africa	Registration (HPCSA)	Graduation from approved Masters degree program plus 2 years of internship (of which one must be post-degree). Submission of an internship portfolio; Logbook (minimum of 200 cases of which at least 24 must have been counselled under supervision); 3 case reports; 1 reflective essay; evidence of research experience; evidence of discipline specific knowledge; training/ supervisor reports; training facility exit assessment.	No	No	Q 2 years by continuing education. A percentage of education must pertain to ethics, human rights or health law.

**Table 3:** Relevant websites of professional genetic counseling organizations and/or for genetic counseling licensure and regulation

Country	Professional organization	Website
International	Transnational Alliance of Genetic Counseling (TAGC)	http://tagc.med.sc.edu/
United States of America	National Society of Genetic Counselors (NSGC)	www.nsgc.org
	American Board of Genetic Counseling (ABGC)	www.abgc.net
	Accreditation Council for Genetic Counseling (ACGC)	www.gceducation.org
	Association of Genetic Counseling Program Directors (AGCPD)	www.agcpd.org
Canada	Canadian Association of Genetic Counseling (CAGC)	www.cagc-accg.ca
Cuba	NCMG	www.instituciones.sld.cu/cngm/
Europe	EMBG (EU GC registration) ESHG	https://www.eshg.org/408.0.html https://www.eshg.org/home.0.html
France	Association of French Genetic Counselors (AFCG).	www.af-cg.fr
The Netherlands	Dutch Association of GC (NVGC); of Clinical Genetics (VKGN); of PAs (NAPA); and of nursing specialists (Venvnvs)	www.NVGC.info/ www.VKGN.org/ www.NAPA.nl www.venvnvs.nl
Portugal	Association of Genetic Counselors (APPAcGen)	http://www.appacgen.org/
Romania	Romanian Association of Genetic Counseling (RAGC)	http://www.geneticcounseling.ro/
Spain	Spanish Society of GC (SEAGEN)	http://www.seagen.org
Sweden	Swedish Society of Genetic Counselors (SSGC) Swedish Society of Medical Genetics (SFMG; including certification for GCs)	Www.sfgv.n.nu Www.sfmg.se
United Kingdom	Association of Genetic Nurses and Counsellors (AGNC) Genetic Counsellor Registration Board (GCRB)	http://www.agnc.org.uk/ http://www.gcrb.org.uk/
Middle East		
Israel	The Israeli Association of Genetic Counselors (IAGC)	http://genetic-counselors.org.il/
Saudi Arabia	Saudi Commission for Health Specialties (SCHS)	http://www.scfhs.org.sa/en/pages/default.aspx
Australia & New Zealand	Human Genetics Society of Australasia (HGSA)	https://www.hgsa.org.au/education-training/genetic-counseling
Asia	Professional Society of Genetic Counselors in Asia (PSGCA)	www.psgca.org
China	Chinese Board of Genetic Counseling (CBGC)	http://www.cbgc.org.cn/
Japan	Japanese Society of Genetic Counseling (JSGC)	http://www.jsgc.jp/ (Japanese language only).
	Japanese Society of Human Genetics (JSHG)	http://jshg.jp/e/index_e.html
	Japanese Board of Genetic Counseling (JBGC)	http://plaza.umin.ac.jp/~GC/
India	Board of Genetic Counseling of India (BGC)	http://www.geneticcounselingboardindia.com/index.html
Phillipines	University of Phillipines, Manila	http://ihg.upm.edu.ph
Taiwan	Taiwan Association of Genetic Counseling (TAGC)	http://www.taiwangc.org.tw
Africa		
South Africa	SASHG (of which GC-SA is a focus group)	http://www.sashg.org http://sashg.org/about/focus-groups/genetic-counselling-south-africa/
	HPCSA	http://www.hpcsa.co.za/PBMedicalDental/Registration

## The Americas

### United States of America (~4000 genetic counselors; 39 Master’s training programs)

Master’s degree trained GCs first originated in the US in 1969 [1, 8]. There are several US-based professional organizations addressing the profession, training, accreditation, and certification (Table 3). NSGC provides a professional status survey with information about areas of clinical and non-clinical practice, salary and roles, and other demographics. There are >1200 applicants yearly to US and Canadian programs (personal communication, Angela Trepanier on behalf of AGCPD) and nearly 400 graduates/year. Master’s training in ACGC accredited programs involves a combination of scientific and counseling coursework, supervised clinical training of no less than 50 cases, and a research project. ACGC graduates are eligible for the ABGC certification exam (Table 2). Starting in 2018, internationally trained GCs can be considered for eligibility to take the exam. Upon graduation and certification, GCs can practice clinically without direct supervision by physicians (though most work in an interdisciplinary environment). Within the US, many professions (including GCs) are regulated on a state-specific basis (called “licensure”), through laws meant to provide consumer protection from harm that may occur due to unqualified individuals providing services. This also provides title protection for licensed professionals. In 2018, 22 (of 50) states license GCs, typically on the basis of ABGC certification. Most employers, even in states without licensure, require either ABGC certification or its eligibility for clinical employment. NSGC has developed a scope of practice for genetic counselors, and in some states licensed GCs are able to order genetic tests independently. There has been significant growth in genetic counseling positions over the past 5 years, now resulting in a workplace shortage that is being addressed in various ways [9] including the expansion of training programs and numbers of enrolled students, and consideration of ways in which GCs can “work at the top of our scope” (of practice) and where our unique skills are most valuable within the changing landscape of genetics and genomic testing.

### Canada (~350 genetic counselors; 5 Master’s training programs)

The Canadian Association of Genetic Counseling (CAGC), currently estimates that most of the ~270 practicing GCs are in clinical genetic counseling roles, with fewer in non-clinical research or industry roles. Training is based on the genetic counselling competencies as defined by CAGC and/or ACGC (which overlap significantly) [10, 11]. There are nearly 200 applicants/year, with 20–25 trainees enrolling each year. Canadian GC graduates are eligible to sit for the CAGC and ABGC certification exams and internationally trained GCs who wish to apply for CAGC certification may inquire as to eligibility; most employers require certification by CAGC or ABGC. Upon graduation and certification, GCs can practice without direct supervision but not autonomously. There is not yet licensure, registration, or other legal recognition of the profession (including title protection) for GCs in Canada due to the lower numbers and cost of such regulatory processes, although three provinces are actively pursuing this.

### Cuba (~900 genetic counselors; 1 Master’s training program)

Cuba is the only country in Latin America that has a profession of GCs [12, 13]. A single Master’s degree program (Havana) is certified by the Ministry of Higher Education. Some trainees are physicians in other specialties, and others (nurses and psychologists) are trained specifically to enter the profession of genetic counseling. All receive training as GCs under the guidance of a Clinical Genetics specialist from the National Network of Medical Genetics. GCs work in community health centers, provincial health centers, and academic (research and reference) hospitals across Cuba.

### Central and South America

Genetic counseling is not yet recognized as an independent profession in Central and South America, but instead is considered a medical competency provided by physicians, mostly geneticists and other specialty physicians (primarily in oncology). Still, the importance of genetic counseling services is highlighted by clinical guidelines from several Latin American countries [14–17]. There are a few formally trained GCs involved in clinical service (personal communication, Sonia Margarit), which is slowly increasing awareness and creating a space and demand for genetic counseling services. As of 2017, there is a single Master’s degree program starting in Sao Paolo, Brazil (see http://www.ib.usp.br/mpag/ accessed May 5, 2018). The limited training and minimal regulation to define the credentials and roles of GCs results in a slow recognition of GCs as profession.

## Europe

The profession of genetic counseling is established or developing in more than 11 European countries, with GCs trained in other countries beginning to establish genetic counseling-related roles in at least 7 more. There are at least eight GC training programs. Formal GC registration occurs through two different programs: in the United Kingdom (UK, via the GCRB as described below) and the European Union (EU) (via EBMG) [18].

### United Kingdom (~310 genetic counselors; 3 Master’s training programs)

Genetic counseling began in the UK in 1992 [19], and the UK currently trains ~40 GCs per year. Historically in the UK, genetic counseling training was a 2-year Master’s degree at a program accredited by the GCRB, followed by a 2-year paid internship and voluntary registration by the GCRB. The AGNC and GCRB have been seeking statutory regulation for a number of years and at the time of writing, statutory regulation, answering to Parliament, is likely to become a reality during 2018.

Since September 2016, there is a funded scientific training program (STP) Master’s in Genomic Counseling for ~10–20 students per year for England only. This 3-year, full time Master’s degree, assessed as equivalent to the previous approach, consists of academic teaching (centralized at the University of Manchester), plus clinical training within a Training Genetics Centre. A new part-time, 3-year, largely e-Learning MSc in Genetic and Genomic Counselling was initiated in 2017 at Cardiff University in Wales. It will accept 20–25 students per year, all self-funded. There is also a Master’s in Genetic Counseling in Glasgow, accepting ~5 students per year, this is also self-funded. GCs had never previously been part of National Health Service (NHS) workforce planning, and the new STP program addresses this for England. Graduates from the STP program will be awarded statutory regulation as clinical scientists, attached to the Academy for Healthcare Scientists and the Health Care Professions Council [20].

Many aspects of the training and registration process for GCs in the UK are still evolving. While GCRB registration (Table 2) is still voluntary, UK employers typically only employ registered GCs or those working towards registration. Internationally trained GCs from some countries can be considered for registration in the UK. Most GCs in the UK work in NHS regional genetics centers, with a few working in research, education, patient support groups, and private clinical practice. In the UK, GCs are qualified to work autonomously or as part of multi-disciplinary team. As genomics is mainstreamed across whole healthcare services, GC roles are evolving [21–23].

### EU registration via EBMG

The Genetic Nurse and Counsellor Professional Branch of the European Board of Medical Genetics (EBMG) developed competencies and standards of practice for GC registration within the European Union (EU) [24, 25]. Given the diversity of languages, healthcare systems, and cultural differences, as well as nationally accepted roles for GCs, it was challenging to develop a single set of standards. A European registration system for GCs was launched in 2013, and is separate from the UK registration process. The EBMG registration recognizes a Master’s in genetic counseling (MSGC) as the “gold standard” for training of GCs, but acknowledges that there are limited MSGC programs in Europe, and many longstanding professional GCs who trained in other ways. The registration system [26] offers evaluation and accreditation of MSGC training programs within Europe. During the first five cohorts of applicants, professionals with longstanding practice as GCs but without Master’s level training were allowed to apply for registration via a grandfather clause that necessitates a larger portfolio of evidence. Internationally trained GCs from the US, Canada, South Africa, Australia with current registration and/or certification in their home country may be considered after working full time in Europe for 1 year. As of 2017 there are 69 EBMG registered GCs/ genetic nurses. A survey of registered GCs showed that they value the confirmation of their competence and to advance the genetic counseling profession in Europe [17]. Recently, the professional branch opened “Associate Registration” in response to requests for a registration system for professionals outside Europe in countries where no registration or certification exists.

### Belgium (>10 genetic counselors; no training programs)

The profession of genetic counselor is not yet recognized in Belgium, and there is not yet any registration process. The genetic counselors practicing in Belgium have been trained outside of Belgium, some but not all at MSGC programs.

### Denmark (>20 genetic counselors; no training programs)

In Denmark most GCs have a professional degree in nursing or laboratory technology or were trained as medical secretaries, and have been trained for genetic counseling at a local clinical genetics department. Some GCs have further education such as a Diploma of Health (60 ECTS), but there are no GCs in Denmark with EBMG registration or an MSGC, although there are efforts to formalize GC education. All of the GCs work in the public health system, primarily in cancer genetics. The clinical roles include case preparation, conveying genetic testing information and working alongside other genetics professionals. There is no specific professional organization for GCs in Denmark, but they typically meet once a year as part of the Danish Society for Medical Genetics for a 1 day continuing education meeting.

### Finland (genetic nurses, no training program)

There are 20 genetics nurses in Finland, ~5 of whom provide genetic counseling services across a range of clinical areas including prenatal diagnosis, carrier status and rare genetic disease, and predictive testing for neurology and oncology. There was previously a genetic nurse training program, and others obtained genetics training on the job. None of the genetic nurses in Finland have EBMG registration.

### France (>175 genetic counselors; 1 Master’s training program)

The profession of GC was legally recognized by French law of Public Health in 2004, and there are up to 20 trainees each year who are eligible for EBMG registration. Nearly 25% of trainees enter with a prior professional degree in nursing, midwifery, clinical research, or laboratory sciences (PhD), and some also go on to an additional PhD after GC training. GCs in France are mainly employed by the public health service within Medical Genetics Services, and the scope of practice is the same for both Master’s and PhD trained GCs [27].

### Ireland (<10 genetic counselors; no training programs)

Most GCs practicing in Ireland have MSGC training from outside of Ireland, and hold GCRB or EBMG registration. Irish GCs practice in a variety of general genetic counseling roles, speciality areas (cancer genetics), and non-clinical roles. GCs are not currently recognized by the Health and Social Care Regulatory Council in Ireland (CORU), but they are in the process of uniting to form a professional association and to obtain professional recognition and regulation.

### The Netherlands (55 genetic counselors; 4 Master’s PA training programs)

GCs training in the Netherlands began in the early 1990s. Early trainees were nurses or other healthcare providers who received on-the-job training by clinical geneticists. This was followed by development of a GC training program in 1997, which was accredited by the VKGN in 1999. Due to an inability to obtain accreditation from the government, a 2015 needs assessment found that a Master’s degree in GC was needed, but the profession was too small to develop a specialized master’s program. As such, in 2016 a decision was made to offer experienced GCs a Master’s degree as a Physician Assistant (MPA) in order to allow GCs to provide services independently from clinical genetics and other physicians. There were two key reasons for this: billing rules in the Netherlands, and that only clinical geneticists and GCs can offer DNA based testing to patients. Four training locations are currently available (Nijmegen, Groningen, Amsterdam, Utrecht). GCs typically work in university hospitals or private outpatient clinics independently. Some GCs are eligible for EBMG registration through the ‘grandfather clause.’

### Norway (40 genetic counselors; 1 Master’s training program)

Norway’s single training program (Bergen) includes coursework and a 1-year research project, but clinical training is not strongly emphasized, and therefore the most important clinical training occurs after employment as GCs. Because of this limited clinical training within the program, GCs in Norway must apply for EBMG registration via the Grandfather Clause until program revisions occur in 2019, when clinical training should meet EBMG criteria. Most entering students have a professional degree in nursing or medical laboratory technology or other relevant education and working experience, a few obtain an additional PhD-degree in genetic counseling. In Norway, The Act of 5 December No. 100 2003 (the Biotechnology Act) frames the regulatory politics concerning medical use of biotechnology. It requires that genetic testing and genetic counseling only occur at approved institutions, which limits genetic counseling to clinical genetics departments at four regional state hospitals. Although this law stresses that genetic counseling should only be performed by trained professionals, genetic counseling as a profession has not yet been authorized. Finally, in Norway there is an “Interest Organization for Genetic Counselors in Norway”, which meets yearly.

### Portugal (<10 genetic counselors; 1 Master’s training program, currently inactive)

The Association of Genetic Counselors (APPAcGen), is working to achieve recognition of the profession of GCs in Portugal. There is a single training program (Porto), which has run only one course (2009–2011) and graduated six GCs at that time. GCs are eligible for EBMG registration [28].

### Romania (>75 trained genetic counselors; 1 Master’s training program, currently inactive)

The Romanian Association of Genetic Counseling (RAGC) was founded to set national practice standards and lobby for recognition as a distinct health profession. In the meantime, genetic counseling tends to adhere to international organizations’ guidelines (e.g., EBMG). Most trainees have prior professional degrees in psychology, nursing, midwifery, or laboratory sciences. Training is structured similarly to the other EBMG approved programs in Europe, and graduates are eligible for EBMG registration after an additional clinical placement. Registration is voluntary and employers do not routinely verify proof of registration. Since the profession is not yet recognized as a distinct health profession, there are few jobs in the public sector and most graduates work in research, education, patient support groups, and private practice.

### Spain (70 genetic counselors; 1 Master’s training program)

The Master’s degree training program (Barcelona) accepts 10–12 students every other year, and some students also pursue a PhD. Clinical genetic counseling jobs are possible in both the public and the private health system, but are more difficult to obtain in Spain as the profession has not yet been recognized and job titles often differ from “genetic counselor.”

### Sweden (~40 genetic counselors; no training programs)

Most GCs in Sweden work in clinical genetics clinics, with some in obstetrics, cardiology or oncology. In the past there have been half-time 2-year programs that included coursework in clinical genetics, ethics and counseling training. The SFMG and SFGV recently developed an educational pathway aiming to certify GCs for clinical genetics work in Sweden [29].

### Switzerland (<10 genetic counselors; no training programs)

Aside from a handful of GCs working primarily in laboratory settings in German-speaking Switzerland, GCs work clinically only in French-speaking Switzerland (Fribourg, Geneva, Lausanne). All have MSGC training from outside of Switzerland. These health professionals work in multi-disciplinary teams located both in the public (hospitals, universities) and in the private (clinics, laboratories) domains. They practice in various fields including oncology, cardiology, neurology, obstetrics, and general genetic counseling (positive family history, cystic fibrosis, hemochromatosis). There is to date no federal recognition ruling on a single and consistent salary scale, and job titles vary.

### Other countries in Europe

There are EBMG registered GCs in Greece, Iceland, Italy, and Belgium and we are aware of a handful of additional MSGCs in Austria [30] and Germany. A 2-year Master's Degree in "Genetic Counselors and Nurses" started in Siena, Italy in February 2017. Genetic counselors are not yet recognized as a profession in Austria, Belgium, Germany, and Portugal (and perhaps other countries) due to legal restrictions requiring that genetic counseling is medical act, and therefore conducted by physicians. In most other European countries where GCs do not provide clinical services, the roles appear quite varied, with some working in laboratories or private companies, providing healthcare provider education or assisting with laboratory reports and genetic test ordering.

## Middle East

There are around 100 GCs practicing in the Middle East, mostly located in Israel and Saudi Arabia; we are aware of a handful of MSGCs in Lebanon, Oman, and Turkey as well.

### Israel (80 genetic counselors; 3 Master’s training programs)

There are currently three 2-year master’s programs in genetic counseling in Israel; curriculum includes coursework, clinical training, and an optional research project (primarily performed in research labs, with a few epidemiological or psychologically based projects). About a third of counselors graduating from the master's program continue their studies for a PhD degree, either directly after graduation, or after a few years of working as GCs. GCs with a PhD have similar roles compared to counselors holding a master's degree, with some having more supervision and teaching roles [31]. In Israel, GCs are licensed (Table 2) and must work under the supervision of a medical geneticist; most counselors work in hospital based genetics departments across the country. A few counselors work in non-clinical research, or in the industry.

### Saudi Arabia (20 genetic counselors; 2 Master’s training programs)

Saudi Arabia had a high diploma (postgraduate) program in Genetic Counseling from 2005 to 2007 [32]. Master’s programs have existed since 2015; both programs include coursework with a focus on genetic counseling in Islam and clinical training. All counselors practicing in Saudi Arabia are expected to obtain a license from SCHS [33, 34].

### Australia/New Zealand (220 genetic counselors; 2 Master’s training programs)

Prior to 2008, GCs in Australia received 1 year of training and a graduate diploma. Since 2011, GC training has required a 2-year Master’s degree [3]. As of June 2017, there are two Master’s level training programs in Australia (Sydney and Melbourne) with no training programs in New Zealand. Tracking data suggests there are several hundred applicants yearly, with ~24 enrolling each year. Upon graduation from an HGSA accredited Master’s program, GCs can practice as Associate Genetic Counselors, working under the supervision of a clinical geneticist (MD) and certified genetic counselor. After 1 year of practice, they are considered board eligible and can enroll in the HGSA certification process. There is a process for international applicants to apply for certification.

Most GCs in Australia practice in hospital settings (both public and private), and increasingly in private practice with clinicians; some GCs are taking on non-clinical roles in laboratories, industry, policy within government, advocacy, or research roles. Others complete a research PhD in a related field to allow them to pursue clinical research. Australia does not yet formally recognize the profession of genetic counseling as an allied health profession within the country. However, as of 2017 Australasian GCs are working towards professional recognition via a nationally approved process of self-regulation. Currently a maintenance of professional standards program is voluntary, but will become compulsory once self-regulation is achieved. A paper by Barlow-Stewart et al. [35] describes many of the workforce issues in New South Wales, giving an overview of many of the broader issues in Australia.

## Asia

Genetic counseling is an emerging profession in most of Asia, and in many countries the profession of GC refers to physicians with expertise in genetics who provide genetic counseling services rather than a specific independent profession. The major professional organization is the PSGCA, which formed in 2015 [36]. They welcome new members, especially GCs practicing in Asia and those from Asia working in other countries.

### China

To date, the China Ministry of Health has not yet recognized GCs as an independent healthcare occupation. There are no official statistics for the number of healthcare professionals (e.g., physicians, nurses, lab technicians) who provide genetic counseling services in China. Genetic counseling is primarily provided by pediatricians or obstetricians, and the utilization of clinical genetic services is low. Many genetic tests can only be performed in academic institutions as research tests or in commercial direct-to-consumer companies for non-clinical use [37]. The Chinese Board of Genetic Counseling was established in 2015 and provides training through short term online and in-person lectures, educational conferences, and certification for trainees. In 2013, Fudan University established the first genetic counseling pilot training program in China with the goal of developing a master’s degree program [38]. However, there are no graduates of this program currently practicing as specialty-trained geneticists or GCs [39].

### India

Currently, genetic counseling is not only provided by individuals trained as GCs, but is also performed by various medical and allied health professionals (clinical and human geneticists, physicians with continuing education in genetics, and medical social workers), prompting the need for a standardized approach to genetic counseling. In an effort to add legitimacy to the profession and maintain professional standards, the Board of Genetic Counseling, India [BGC] was established in 2014 [36]. There is a growing trend among the graduates trained in India to join the genetic diagnostics industry in genetic counseling roles as well as test reporting, sales, marketing, or medical science liaison roles. Currently, the BGC does not accredit training programs. There are 3 types of genetic counseling training in India: (1) a 2-year Master’s program at Kasturba Medical College, Manipal, (2) a post-graduate 1-year diploma program at Kamineni Hospital, Hyderabad, and (3) a 1-year certificate program at Manipal Hospital, Bangalore [40]. There are additional genetic counseling modules and workshops offered by universities, physician associations and diagnostic testing companies. A handful of Master’s trained GCs (US, UK, and Australia) contribute to the profession through industry, consultancy, clinical roles and pedagogical contributions to the existing educational programs.

### Japan (~230 genetic counselors, 14 Master’s training programs)

The first training program in Japan was started in the early 2000’s. Curriculum and clinical training requirements are based on the US training model, although all program directors are physicians, and psychologists are used to teach the psychosocial curriculum in most programs. There is a national certification process; international graduates may request an application for certification examination. GCs in Japan work in cancer genetics, prenatal, pediatric, and neurogenetics areas, as well as more general genetic counseling positions, and are mostly employed in larger cities at larger (often academic) medical centers or in private practice.

### Malaysia (<10 genetic counselors, 1 Master’s training program)

Genetic counseling services began in 1994 [41] and GCs in Malaysia have MSGC training from outside of the country as there is not yet any formal genetic counseling training in Malaysia. GCs provide genetic counseling to patients at academic universities and cancer research centers, and through diagnostic companies; one GC is in private practice. In 2015, the National University of Malaysia (NUM) launched a Master’s in Medical Science (Genetic Counseling) to train more GCs in Malaysia [42]. Plans are in progress to form a professional society, to create practice guidelines and certification pathways for all GCs working in Malaysia.

### Philippines (>10 genetic counselors, 1 Master’s training program)

Genetic counseling training includes clinical training and a research requirement, has a significant focus on public health, including rare disorders and newborn screening. Many of the entering students have prior background in the sciences or various healthcare professions. Education is provided in English and Filipino, taking into account the many dialects across the Philippines, and funding is available for individuals from diverse regions to attend the program and return to their province in order to provide services countrywide. GCs in the Philippines are involved in various clinical activities, research and academic endeavors.

### Singapore (10 genetic counselors, no training programs)

The genetic counseling profession in Singapore is still developing; the job description and scope of practice is not formalized across the country and the role is not yet recognized as an Allied Health profession. MSGCs trained in countries outside Singapore provide clinical genetic counseling in cancer genetics, general medical genetics (pediatrics/adult) and prenatal genetics, and research roles. The National Cancer Centre Singapore (NCCS) is spearheading efforts to develop GC-led clinics and paired GC-geneticist clinics, modelled on the NSGC scope of practice. This model has resulted in improved efficiencies and cost-savings [43].

### South Korea (12 genetic counselors; 2 Master’s training programs)

There are 2 Master’s level training programs (Suwon, Daejeon), and certification is provided by the KSMG [44].

### Taiwan (120 genetic counselors, 1 Master’s training program)

A single 2-year master’s degree program (National Taiwan University) requires at least 300 internship hours (clinical and laboratory) prior to certification. Trainees are recruited from baccalaureate backgrounds including life science or related field, such as medicine, nursing, or medical technology. GCs work genetic counseling centers, private prenatal service providers, laboratories or other related organizations [45].

## Africa

### South Africa (~20 genetic counselors, 2 Master’s training programs)

Currently in Africa, there are medical genetics services in many countries, but the only formal genetic counseling services and training programs are in South Africa . Genetic counseling training started in 1989 in South Africa, and there are currently two 2-year master’s degree training programs (Johannesburg, Cape Town) [46, 47, 48]. Clinical training requires a 2-year internship, with the first year overlapping with the second year of the degree. Paid internships are challenging to obtain. GC practice is governed by the HPCSA, and GC-SA developed the standards of practice guidelines to guide the training and registration of GCs in alignment with the regulations approved by the HPCSA. In order to practice clinically, a GC must be registered through HPCSA, who registers GCs under the category “Medical Scientists” under the Medical and Dental Board. Internationally trained GCs from the UK, Europe and Australia are eligible for registration; GCs from other countries are considered on a case by case basis. Genetic counseling jobs are primarily clinical, in urban areas, in both state hospitals and private services, and recently expanding into industry.

### Summary

We believe that in 2018 there are nearly 7000 genetic counselors globally, in at least 28 countries. Greater than 60% are practicing in North America. As described above, most countries with training programs in genetic counseling have adopted a 2-year Master’s degree approach, although there is variation in methods and requirements for training, certification, and registration/licensure. Our data suggests that flexibility in how the profession develops and expands will remain necessary to acknowledge the international variation in laws, health systems, and cultures. Genetic counseling remains a growing profession globally, and international collaboration and growing reciprocal agreements support ongoing improvements across training, regulation, and scopes of practice.
